# Associations between structural holes in personal networks and health behaviors among young and middle-aged adults in Japan: a population-based cross-sectional study

**DOI:** 10.3389/fpubh.2025.1621420

**Published:** 2025-09-03

**Authors:** Yasutaka Kuribayashi, Daisuke Takagi

**Affiliations:** ^1^Department of Health and Social Behavior, Graduate School of Medicine, The University of Tokyo, Tokyo, Japan; ^2^Department of Social Impact Assessment, Graduate School of Medicine, Kyoto University, Kyoto, Japan

**Keywords:** Japan, social network, structural hole, health behavior, diffusion stage, egocentric network

## Abstract

Previous studies have emphasized that tightly knit networks influence health behaviors. However, effective network structures for behavioral adoption may vary by diffusion stage. This study examines how the association between personal network structures and health behaviors varies across behaviors with different prevalence degrees. We used data from the third-wave Japanese Study on Stratification, Health, Income, and Neighborhood (J-SHINE) conducted in 2017, targeting residents aged 32–58 years in Japanese metropolitan areas. Peer characteristics, behaviors, and interconnections were collected using the name generator method. Data from 1,705 respondents (egos) and 6,820 peers were analyzed. Structural holes, as the network structural characteristic, were evaluated using the reciprocal of the dyad constraint index of each ego-peer pair and categorized into tertiles. Logistic regression analyses examined the associations of structural holes with ego’s exercise and preventive dental care use (intermediate prevalence stage) and non-smoking behavior (later prevalence stage), adjusting for covariates. Results showed that, compared to peers with middle-level structural holes, those with many structural holes were positively associated with ego’s exercise habits (odds ratio [OR], 1.35; 95% confidence interval [CI], 1.19–1.52) and preventive dental care use (OR, 1.20; 95% CI, 1.07–1.35), while peers with few structural holes were negatively associated with ego’s non-smoking behavior (OR, 0.81; 95% CI, 0.70–0.94). The findings suggest that the association between structural holes and health behaviors varies according to the diffusion stage. Considering social connections with different levels of structural holes by diffusion stage of the target behavior may be effective for public health interventions.

## 1 Introduction

### 1.1 Background

Social network studies have demonstrated that health behaviors, including health-promoting and risk behaviors (1), are affected by the social networks in which individuals are embedded (2–5). Conceptual models explain that the structural and relational characteristics of social networks influence health behaviors through social support and social influence (6). For instance, peers and family members can strongly influence the decision to seek medical care by providing informational support (7–9). Social influence implies that connection with others affects one’s behavior through normative pressure or as a source of social learning (6). Previous studies have shown that network members influence health behaviors, such as smoking (4, 10), alcohol consumption (11, 12), and contraceptive use (13) through social influence and learning.

### 1.2 Theoretical mechanisms relating network structure and health behaviors

Recent studies applying network analysis techniques have suggested that network structure is critical for determining how health behaviors spread in the network. Existing literature shows that the influence on the diffusion of health behaviors varies depending on the structural positions (i.e., centrality) of other members within a network (10, 14). However, what type of network structure is most effective in promoting health behaviors remains controversial (15).

According to existing social network theories, there are two contrasting arguments regarding the relationship between network structure and the spread of health behaviors. One argues that dense networks with redundant ties facilitate the spread of behavior by exerting social influence. For example, in an experiment using online social networks, Centola (16) demonstrates that health behaviors (participation in a health forum) were more likely to spread within clustered rather than non-clustered networks. Similarly, a survey on the large-scale distribution of deworming drugs across 17 villages in Uganda revealed that interpersonal communication was facilitated in villages with clustered networks of residents and community medicine distributors. This helped enhance the reach and speed of drug administration (17). These studies suggest that, unlike simple contagions like diseases, health behaviors spread through multiple exposures to information and others’ behaviors, thereby making social influence in clustered networks advantageous.

Conversely, other theories argue that clustered networks are not always beneficial. Instead, sparse networks without redundant ties are more suited to behavior spread. Granovetter (18) distinguished strong and weak ties based on contact frequency, emotional intensity, closeness, and reciprocal service. His “strength of weak ties” theory suggests that weak ties, although not closely connected to the focal individual or their friends, bridge different groups and provide access to new and diverse information. A study of deprived areas in England showed that heterogeneous and weak-tie networks expanded the range of accessible resources, which may lead to health benefits (19). However, instead of the structural aspects of networks, the “strength of weak ties” theory focuses on the characteristics of individual connections. Burt’s “structural holes” theory, considering network structural characteristics, addresses this gap (20). Structural holes represent an absence of social ties between peers. When peers do not know each other, they are likely to belong to different social groups, each serving as a distinct source of information and perspective for the focal group (21). For example, a study of middle-aged female sex workers in China showed that those embedded in networks with many structural holes were more likely to access diverse social support than those in tightly knit networks (22). Similarly, a study of older adults in the US found that those with many structural holes in their personal networks reported increased access to a broader range of information and a high likelihood of using alternative medical services (23).

Previous studies have examined the influence of both many and few social ties within a network on the adoption of health behaviors. However, the effective features of network structures for behavioral adoption may differ depending on the behaviors, which remains unexplored.

### 1.3 Diffusion of health behaviors through social network structures

Studies on the diffusion of innovation demonstrate that new ideas and practices typically spread from outside the community to the inside (24). Actors and information sources outside the community play a critical role in the early diffusion stages, whereas internal communication within the community becomes more influential in the later stages. For example, a study on the diffusion of global tobacco control treaty ratification suggested that external sources of information were essential in the early stages, whereas as diffusion progressed, internal sources of information became more important (25). A simulation study of the diffusion processes also showed that bridging structures, where peers with structural holes connect different communities, allow for the fastest diffusion of behaviors in the early diffusion stages (26). An empirical study in innovation research also demonstrates that open network structures, characterized by structural holes, positively impact individual innovation ability, whereas closed networks impact it negatively (27).

A previous study showed that individuals who adopt innovations can be categorized into five ‘adopter’ categories according to their degree of innovativeness, as assessed by the time at they adopt an innovation. These categories were innovators (2.5% of the total), early adopters (13.5%), early majority (34%), late majority (34%), and laggards (16%). The early majority, comprising 34% of the social system, tended to adopt new ideas and practices slightly earlier than the average member of the system, as opposed to the late majority that adopted them slightly later (24). Based on these findings, the diffusion of behaviors can be considered in three stages: early, intermediate, and later. Up to 16% prevalence, the innovators and early adopters were primarily responsible for adoption, which may correspond to an early diffusion stage. Up to 50%, the early majority contributed significantly, suggesting an intermediate stage of diffusion. Over a prevalence of 50%, the late majority and laggards started adopting innovation, which might indicate a later diffusion stage.

Based on the above discussion, effectiveness of network structures for adopting health behaviors may vary depending on the diffusion stage. For the early diffusion stages, findings from innovation research suggest that network members with many structural holes, who provide access to diverse information and perspectives, are essential for the behavior adoption of focal one. As diffusion progresses, network members who exert social influence—those with many ties within the network—gradually become increasingly important. Therefore, in the intermediate stages of diffusion, networks with many or few structural holes may play important roles. Those with few structural holes become essential in later stages.

In Japan, health behaviors among young and middle-aged adults, including exercise habits and dental check-up, are in the intermediate diffusion stage, with participation rates of 46 and 50%, respectively (28, 29). At this stage, peers with many as well as few structural holes may be crucial. Conversely, the smoking rate has steadily declined to 15% (30). This low smoking rate suggests that non-smoking behavior, which includes former smokers as well as those who never smoked, is in the later diffusion stages. At this stage, peers with few structural holes may be important.

### 1.4 Purpose and hypotheses

This study aims to clarify the association between personal network structures and health behaviors using structural holes as network structural indicators. It examines the following hypotheses:

*Hypothesis 1*: Exercise habits and preventive dental care use, which are in the intermediate stage of prevalence, are positively associated with the presence of peers with many as well as few structural holes in personal networks.

*Hypothesis 2*: Non-smoking behavior, which is in the later stage of prevalence, is positively associated with the presence of peers with few structural holes in personal networks.

If these hypotheses are supported, targeting network structures tailored to the stage of behavior diffusion can help promote healthy behavior.

## 2 Methods

### 2.1 Data

**Figure 1:**
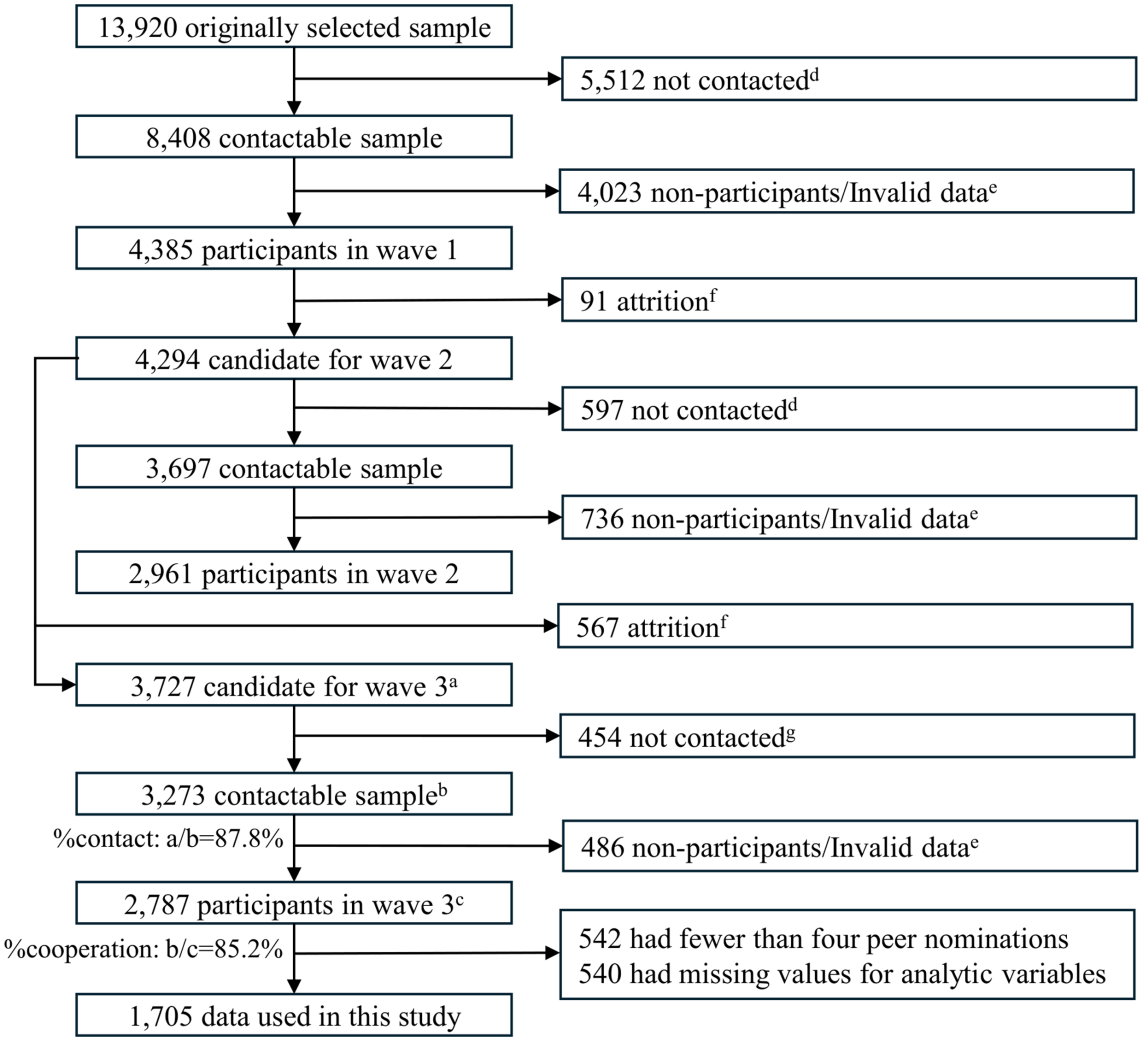
Flowchart of the selection process of participants. d, Death, ineligible age, unidentified address, long-term absence, and inaccessible contact. e, Refusal, break-off, spouse/partner wrongly answered. f, Death, drop out. g, Inaccessible contact, long-term absence.

We used data from the third-wave survey of the Japanese Study on Stratification, Health, Income, and Neighborhood (J-SHINE) conducted in 2017. The J-SHINE project conducted the first-wave survey in four municipalities of the Tokyo metropolitan area (Adachi, Mitaka, Kashiwa, and Tokorozawa) in 2010. Of the 13,920 adults aged 25–50 years probabilistically selected from the Basic Resident Register, 8,408 were contactable, and 4,385 participated. Details of the first-wave survey are documented elsewhere (31). In 2012, the second wave was conducted with 4,294 participants, excluding those who had passed away or indicated permanent refusal. Responses were obtained from 2,961 subjects. The respondents completed self-administered questionnaires using computers in both the first and second waves. In 2017, the third-wave survey was conducted with the participants of the first-and second-wave surveys. Of the 3,727 eligible subjects, 3,273 were contacted (contact rate: 87.8%), of whom 2,787 participated (cooperation rate: 85.2%). The overall participation rate was 74.8% (0.878 × 0.852 = 0.748). In the third wave, participants completed paper-based, self-administered questionnaires distributed by trained surveyors. The surveys were posted to participants who had relocated to other municipalities. Figure 1 presents a flowchart of the participant selection process.

### 2.2 Measurement

#### 2.2.1 Personal social network

**Figure 2:**
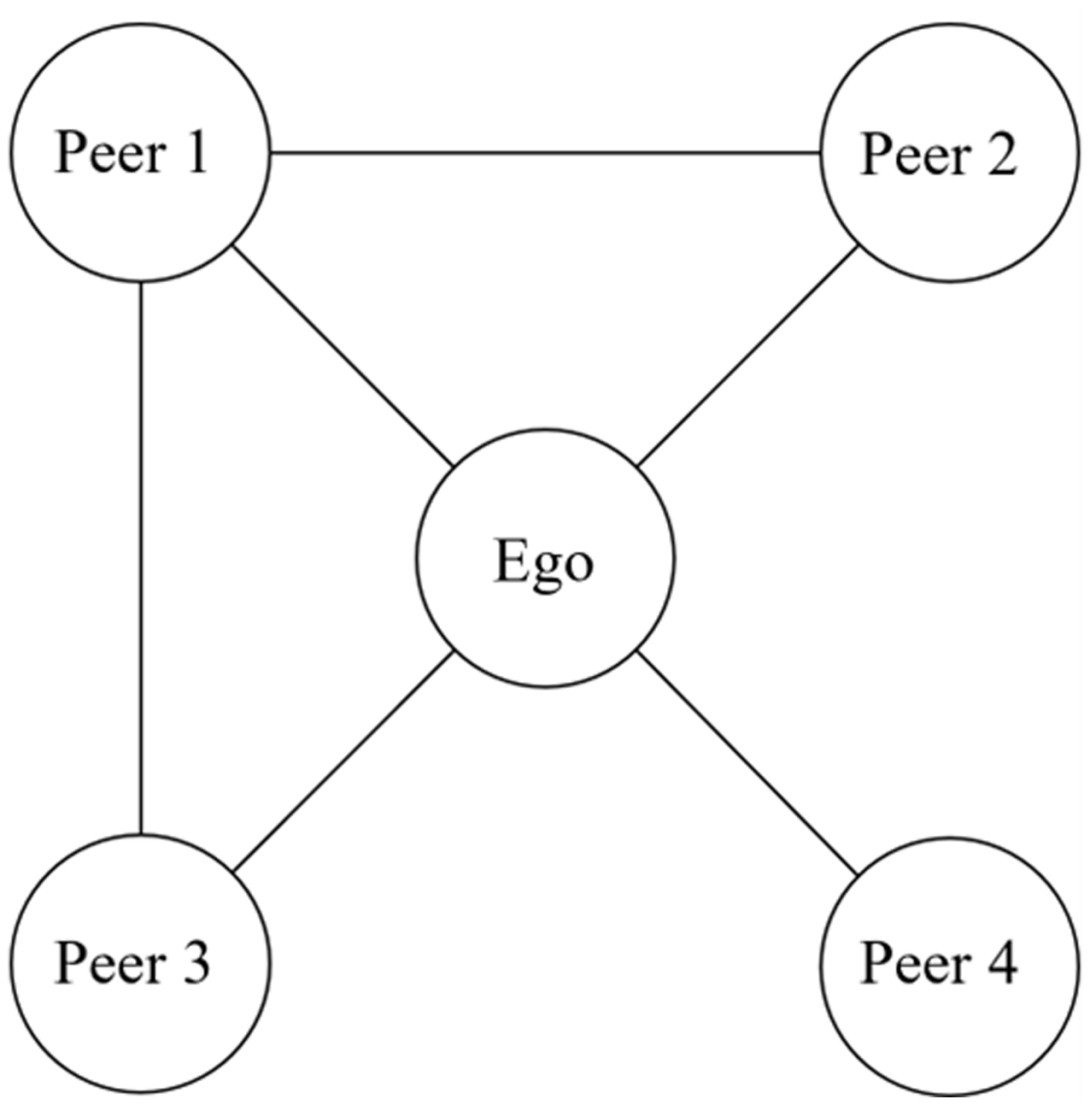
Example of a network.

We employed the name generator and name interpreter methods to measure personal social networks (32). Respondents were asked to name four friends or acquaintances aged 20 years or older with whom they interacted or communicated frequently. To focus on peer-based structural advantages in access to diverse information and perspectives, we excluded immediate family members or relatives from the ego network questions. Based on human time and cognitive constraints, the number of close relationships that substantially influence behavior is estimated to be approximately five (33). However, following previous studies (10, 34) and considering the cognitive load on the respondents in survey-based network research, we limited the number of peers in our name generator questionnaire to four. Following its usage in the field of network analysis, we hereafter refer to the respondent as ‘ego.’ We asked the ego about each peer’s social characteristics, health behaviors, and social ties with peers, as known by the ego. The social ties between the egos and their four peers were represented in a 5 × 5 matrix, where connected and unconnected pairs were coded as 1 and 0, respectively. For example, Figure 2 illustrates the social network of an ego connected to four peers, where Peer 1 is linked to Peers 2 and 3.

#### 2.2.2 Structural holes in the network

Following previous studies, we used a constraint index to assess structural holes (20, 35). The constraint index measured the extent to which the peers directly connected to an ego were interconnected within a network. The constraint index can be calculated for each ego-peer pair as the “dyad constraint index,” based on each peer’s connectivity with other peers. The inverse of the dyad constraint index is treated as the index of the structural holes of each peer, as lower constraint index values correspond to more structural holes (36).

Mathematically, the dyadic constraint that the ego receives from a peer is defined by the following equation (20):


$$C_{\mathrm{ij}}={\left(P_{\mathrm{ij}}+\sum P_{\mathrm{iq}}P_{\mathrm{qj}}\right)}^{2},\;\;q\neq i,j$$

**Figure 3:**
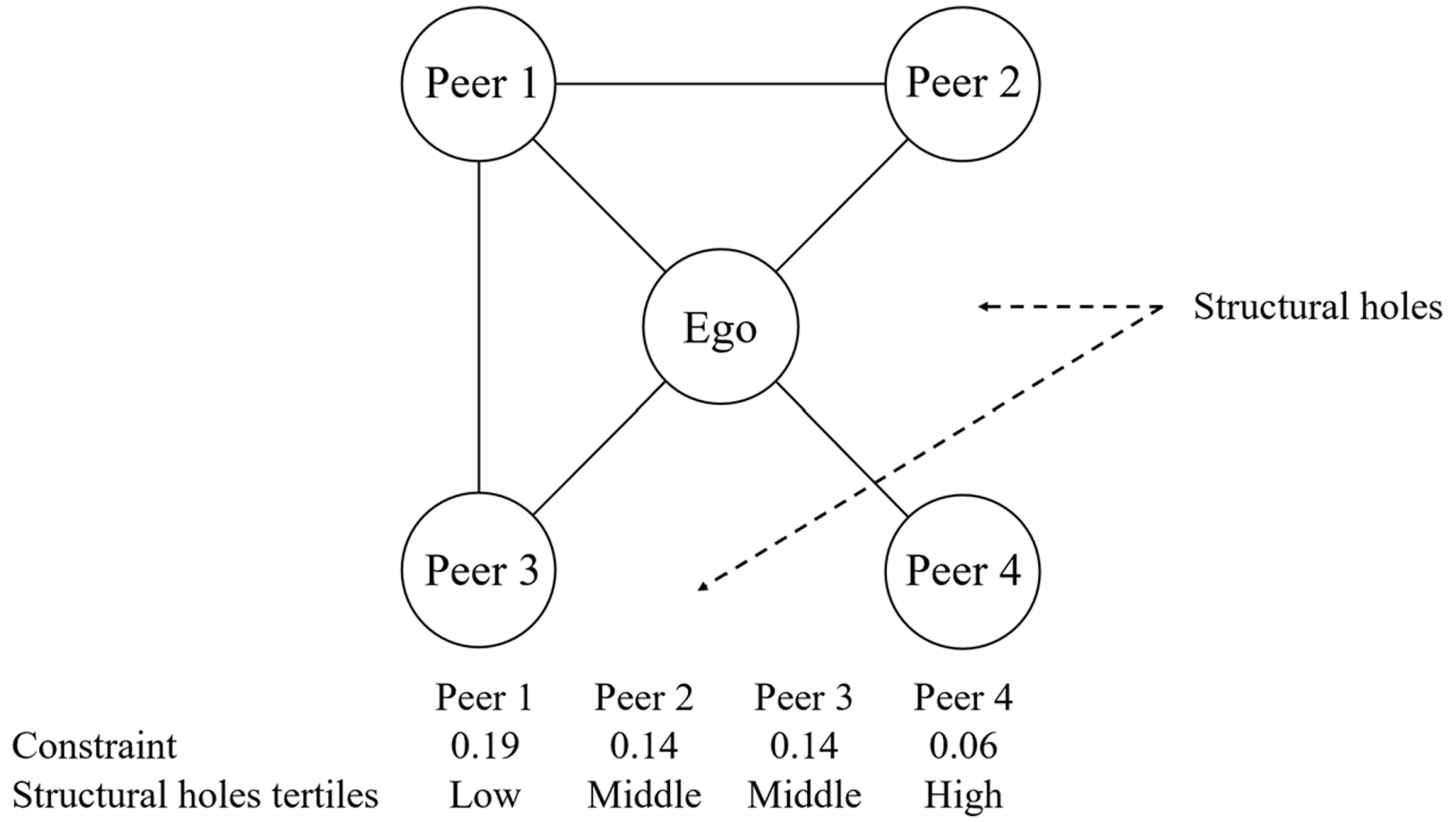
Example of constraint and structural holes in personal networks. The structural holes calculated from the dyadic constraint between each peer and ego were categorized into three groups: “low,” “middle,” and “high,” based on tertiles.

$P_{\mathrm{ij}}$ represents the strength of the tie between ego i and peer j , divided by the total tie strength with all peers. Similarly, $P_{\mathrm{qj}}$ represents the proportional strength of the tie between peer q and peer j . Figure 3 illustrates examples of the constraints and structural holes in an egocentric network. The extent to which peer 1 constrains the ego is the largest, and there are few structural holes between peer 1 and the other peers. On the other hand, the extent to which peer 4 constrains the ego is the smallest, and there are many structural holes between peer 4 and the other peers.

In this study, structural holes were calculated based on the dyadic constraint between each peer and the ego. We examined the distribution of the dyad constraint index and found it to be moderately right-skewed. A histogram illustrating this distribution is provided in the Supplementary Figure 1. To improve interpretability and account for potential nonlinear associations with health behaviors, we grouped each ego-peer pair into three categories based on tertiles of this variable, representing “low,” “middle,” and “high” levels of structural holes. The “high” category of structural holes indicates many structural holes with the peer. Peers in this category are considered to belong to social groups different from those of other network members and tend to bridge different communities (20), facilitating access to diverse information and perspectives for the ego (21).

Because the constraint index varies with network size (22, 37), and it was not feasible to adjust for network size to standardize constraints, we limited our analyses to data from participants with a uniform network size of four peers. Consequently, our analyses included 1,705 egos with 6,820 (=1,705 × 4) peers after excluding those who nominated fewer than four peers (*n* = 542). Participants with missing data on any of the outcome variables or covariates, except for income, were excluded from the analysis (*n* = 540). Overall, sociodemographic characteristics and health behaviors were largely comparable between egos who named fewer than four peers and those who named exactly four peers. However, significant differences were observed between the two groups in terms of marital status, working status, alcohol consumption, and equivalent income (*p* = 0.03, < 0.01, < 0.01, and 0.03, respectively). No significant differences were found for the other variables (Supplementary Table 1).

#### 2.2.3 Ego’s health behavior

Preventive dental care in the past year was self-reported by responding to the following question: “In the past year, have you visited a dentist for dental scaling or fluoride or orthodontic treatments?” Those who utilized preventive dental care were coded as 1 and those who did not were coded as 0. Exercise habits were self-reported through responses to the following question: “In the past year, on average, how many days per week did you exercise for at least 10 min? Please consider only physical activities to improve or maintain health or fitness.” Those who exercised every day, 5–6 days per week, 3–4 days per week, or 1–2 days per week were coded as 1, whereas those who exercised only a few times per month or hardly ever were coded as 0. While the Ministry of Health, Labor and Welfare in Japan defines regular physical activity as engaging in at least 30 min of exercise twice per week for more than a year, our questionnaire did not permit a direct classification of “twice per week.” Instead, participants selected from adjacent response options such as “once or twice per week” or “three to four times per week.” Therefore, we adopted a threshold based on national surveillance data from the Japan Sports Agency, which defines regular activity as exercising at least once per week (28). Smoking behavior was self-reported by selecting one of three predetermined categories: current smoker, former smoker, or never smoked. Current smokers were coded as 0, and former smokers and those who had never smoked were coded as 1.

#### 2.2.4 Covariates

We included covariates based on previous studies to account for potential confounders and other relevant factors associated with health behaviors, thereby improving model comparability. We used the egos’ age, sex, educational attainment, marital status, working status, smoking status, alcohol consumption, equivalent income, place of residence, and peer health behavior score as covariates. Age was considered a continuous variable. Sex, which refers to biological sex, was coded as 1 for men and 0 for women. Information on egos’ educational attainment was obtained from the first and second waves, with code 0 for high school education or lower, including graduates of the upper-secondary division of specialized training colleges (programs requiring junior high school completion for admission), and as 1 for those with university education or higher, including graduates of the post-secondary division of specialized training colleges (programs requiring high school completion for admission) and technology colleges. Marital status was coded as 1 for married and 0, otherwise. Working status was coded as 1 for employed and 0 for unemployed. To analyze exercise habits and preventive dental care use, the egos’ smoking status was further controlled. Alcohol consumption was self-reported through responses to the following question: “In the past year, on average, how often did you consume alcoholic beverages?” Participants who reported drinking every day, 5–6 days per week, or 3–4 days per week were coded as 1, whereas those who reported drinking 1–2 days per week, a few times per month, almost never, or were unable to drink were coded as 0. The equivalent income was calculated using the OECD-modified equivalence scale (38), adjusting household income for household size. For participants with data on individual income but not on household income, the former was used as the equivalent income. Equivalent income was categorized into four groups: “low,” “middle,” and “high,” based on tertile, and “missing” for incomplete data. Because equivalent income had a substantial proportion of missing responses, we created a “missing” category to preserve the analytic sample and acknowledge the potential relevance of income nonresponse. Places of residence were categorized into four municipalities. The peer health behavior score was a composite variable based on peers’ exercise habits, smoking status, alcohol consumption, and obesity, all of which were reported by the ego. Each component was coded as 1 for a healthy behavior (engaging in regular exercise, not smoking, not drinking alcohol, and not having obesity) and 0 for an unhealthy behavior (not exercising, smoking, drinking alcohol, and having obesity). As with other health behaviors, obesity was assessed based on the ego’s subjective evaluation of each peer’s body size. Responses were dichotomized into “underweight or normal” and “overweight or obese.” To retain the full sample and ensure that the index could be computed for all cases, missing values on any of the four binary indicators were coded as 0. We conducted a principal component analysis using tetrachoric correlations among the four binary variables and used the first principal component score as the peer health behavior score in the logistic regression analyses. Previous studies have shown that ego and peer health behaviors are mutually associated (3, 4). To clarify the association between network structure and ego’s health behaviors, we adjusted for the peer health behavior score.

### 2.3 Statistical analysis

Binary logistic regression analyses were conducted. The outcome variables were ego’s exercise habits, preventive dental care use, and non-smoking behavior. The structural holes of each peer constituted the main explanatory variable, with the middle category as the reference. The unit of analysis was ego-peer ties. Therefore, the calculated odds ratios corresponded to each ego-peer tie rather than with the entire personal network. Robust standard error estimates were used to account for clustering by ego (39). All analyses were conducted using Stata 17.0 (StataCorp, College Station, TX, United States). Age and the peer health behavior score were treated as continuous variables, as approximately linear associations with the outcomes were confirmed by preliminary analyses in which these variables were treated as categorical. To assess multicollinearity, variance inflation factors (VIFs) were calculated using linear regression models with the same independent variables; all VIFs were below 2.0, with mean VIFs of 1.3 for preventive dental care, 1.3 for exercise, and 1.3 for non-smoking, indicating no serious multicollinearity. Model fit was assessed using the Hosmer–Lemeshow goodness-of-fit test and pseudo R^2^ statistics. The Hosmer–Lemeshow χ^2^ statistics for exercise habit, preventive dental care use, and non-smoking behavior were 13.1 (*p* = 0.11), 28.4 (*p* = 0.0004), and 23.8 (*p* = 0.0025), respectively, indicating statistically significant deviations. However, this test is known to be sensitive to large sample sizes and may yield significant results even when the model fit is adequate (40). As a complementary measure, we also evaluated pseudo R^2^ values. The McFadden R^2^ values were 0.04 (exercise habit), 0.03 (preventive dental care use), and 0.14 (non-smoking behavior), and the corresponding Nagelkerke R^2^ values were 0.07, 0.06, and 0.22, respectively.

## 3 Results

Descriptive statistics showed that the percentage of egos with exercise habits was 38% (Table 1), which was lower than the 46% observed for the same age group in Japan (28). The percentage of egos using preventive dental care in the past year was 51%, which was comparable to the dental check-up rate of approximately 50% among the same age group in Japan (29). The percentage of non-smokers, which including both former smokers and those who had never smoked, was 79%, which was lower than the 85% observed for the same age group in Japan (30). There were more men than women in terms of peer characteristics, and peers were similar to egos in age, educational attainment, and working status.

**Table 1:** Descriptive statistics for egos and peers.

Egos	*n* = 1,705		Peers	*n* = 6,820	
	n	%		n	%
Age (years), mean (SD)	45.7	6.9	Age		
Male	716	42	Within ± 5 years	3,956	58
Educational attainment			Younger or older than ± 5 years	2,864	42
High-school graduation or lower	444	26	Male	3,956	58
College graduation or higher	1,261	74	Educational attainment		
Marital status			High-school graduation or lower	1,569	23
Married	1,261	74	College graduation or higher	4,092	60
Unmarried	444	26	DK / NA	1,228	18
Work status			Work status		
Working	1,483	87	Working	5,865	86
Not working	222	13	Not working	818	12
Exercise habit			Exercise habit		
At least once per week	648	38	Daily or several times a week	1,296	19
Less than once per week	1,057	62	Several times a month or rarely	2,796	41
Preventive dental care use	870	51	DK / NA	2,728	40
Smoking			Smoking		
Current	245	13	Current	1,432	21
Former	422	23	Non-smoker	5,388	74
Never	1,038	56	Alcohol		
Alcohol consumption			Consumes alcohol	4,774	70
3–4 times per week or more	631	37	Do not consume alcohol	1,364	20
1–2 times per week or less	1,074	63	DK / NA	614	9
Equivalent income	391	306	Obesity		
thousand JPY (/year),			Underweight or Normal	5,719	78
(median [IQR])			Overweight or Obese	1,432	21
Equivalent income categories			Health behavior score, mean (SD)	2.8	1.1
Low	529	31	Constraint, median (IQR)	0.14	0.11
Middle	529	31	Structural holes		
High	426	25	Low	2,251	33
DK / NA	221	13	Middle	2,251	33
Place of residence			High	2,318	34
Adachi	324	19			
Mitaka	357	21			
Kashiwa	512	30			
Tokorozawa	512	30			

Data are presented as n (%) otherwise indicated. SD, standard deviation; IQR, interquartile range; JPY, Japanese Yen; DK/NA, Do not know/no answer.

Logistic regression analyses showed that peers in the high-structural-hole category were associated with a higher likelihood of ego exercise habits (odds ratio [OR], 1.35; 95% confidence interval [CI], 1.19–1.52) and preventive dental care use (OR, 1.20; 95% CI, 1.07–1.35), as compared to those in the middle-structural-hole category (reference category; Table 2). Peers in the low-structural-hole category were not associated with these behaviors. We conducted two sensitivity analyses on exercise habits. In the first, participants who exercised three to four times per week or more were defined as having exercise habits. In the second, participants who exercised a few times per month or more were defined as having exercise habits. Both analyses showed consistent positive associations between structural holes and egos’ exercise habits (Supplementary Table 2). Meanwhile, peers in the low-structural-hole category were associated with a lower likelihood of non-smoking behavior (OR, 0.81; 95% CI, 0.70–0.94). Peers in the high-structural-hole category were not associated with this behavior.

**Table 2:** Logistic regression estimates for each health behavior.

Outcomes:	Exercise habit		Preventive dental care use		Non-smoking behavior	
Explanatory variables	Odds Ratio	95% CI	Odds Ratio	95% CI	Odds Ratio	95% CI
Age	1.03*	1.02–1.04	1.00	0.99–1.00	1.00*	0.99–1.01
Male sex	1.59*	1.41–1.78	0.60*	0.53–0.66	0.29*	0.25–0.33
College graduation or higher	1.20*	1.07–1.36	1.41*	1.26–1.59	2.35*	2.05–2.70
Married	1.01	1.00–1.02	1.00	0.99–1.00	0.99	0.99–1.00
Working	0.77*	0.66–0.90	0.72*	0.62–0.84	0.46*	0.34–0.61
Current smoker	0.64*	0.56–0.74	0.77*	0.68–0.87	—	—
Alcohol consumption	1.21*	1.08–1.34	1.08	0.97–1.20	0.65*	0.57–0.74
Equivalent income						
1st tertile (Low)	1.00		1.00		1.00	
2nd tertile (Middle)	0.99	0.87–1.13	1.17*	1.03–1.32	1.58*	1.35–1.85
3rd tertile (High)	1.49*	1.30–1.71	1.47*	1.29–1.68	1.79*	1.51–2.13
Missing	1.36*	1.15–1.60	1.05	0.90–1.23	1.25*	1.04–1.54
Peer health behavior score	1.42*	1.15–1.62	1.07	0.94–1.22	2.13*	1.80–2.50
Place of residence						
Adachi	1.00		1.00		1.00	
Mitaka	1.12	0.95–1.31	1.01	0.87–1.19	1.48*	1.21–1.81
Kashiwa	1.26*	1.09–1.46	1.11	0.97–1.28	1.76*	1.51–2.13
Tokorozawa	0.99	0.85–1.15	0.89	0.78–1.03	1.18*	1.01–1.41
Structural holes						
1st tertile (Low)	0.97	0.85–1.09	1.06	0.94–1.19	0.81*	0.70–0.94
2nd tertile (Middle)	1.00		1.00		1.00	
3rd tertile (High)	1.34*	1.19–1.51	1.20*	1.07–1.35	0.88	0.75–1.03

95% CIs were based on robust standard errors. CI, Confidence interval. *p < 0.05.

**Figure 4:**
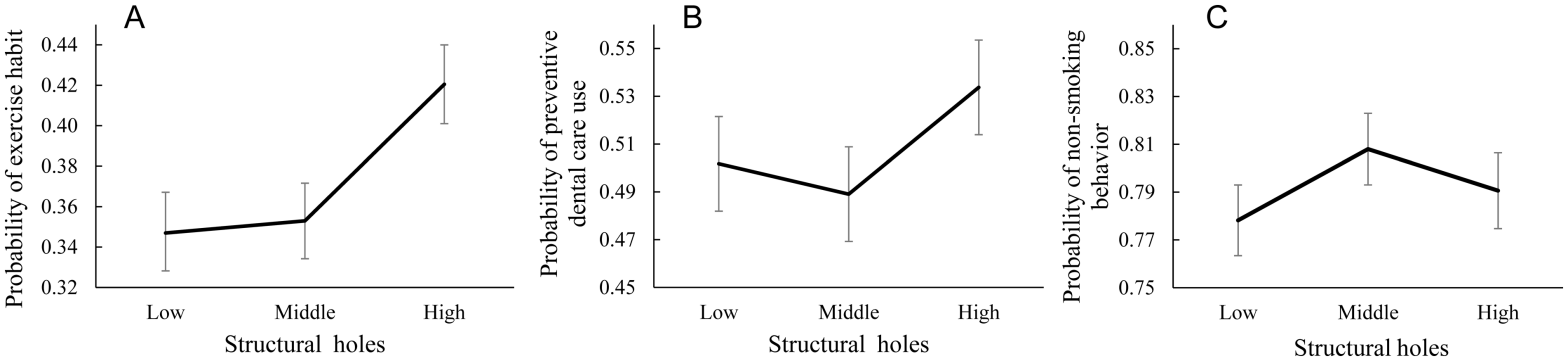
Predicted probabilities of ego’s each health behavior. Error bars represent the 95% confidence intervals for the predicted probabilities. **(A)** Exercise habit. **(B)** Preventive dental care use. **(C)** Non-smoking behavior.

Figure 4 demonstrates the predicted probabilities of ego’s health behavior when peers’ structural holes were “low,” “middle,” and “high,” calculated based on the logistic regression estimates. All covariates were set as mean values. The figure demonstrates that having peers in the high-structural-hole category was positively associated with exercise habits and preventive dental care use. By contrast, non-smoking behavior tended to be positively associated with peers in the middle-structural-hole category.

## 4 Discussion

Previous studies have revealed the advantages, such as in performance and promotion, of a lack of social connections within social networks (i.e., structural holes) in the fields of business and politics (41–43). By contrast, public health studies have often emphasized the influence of many social connections on the adoption of health behaviors (4, 16, 44). However, it is unclear whether the type of health behavior varies among effective network structures for adoption. This study examined the associations between structural holes in social networks and the adoption of different types of health behaviors (exercise, preventive dental care use, and non-smoking behaviors) among young and middle-aged adults in Japan. The results partially supported 1 for exercise and preventive dental care use. Having peers with many structural holes is positively associated with the prevalence of exercise habits and preventive dental care use. Hypothesis 2, regarding non-smoking behavior, was not supported. That is, having peers in the middle-structural-hole category was associated with a higher likelihood of non-smoking behavior than having peers with low levels of structural holes. These findings suggest that the association between peers’ structural holes and ego’s health behavior adoption varies by diffusion stage.

Previous studies have shown that structural holes in personal networks promote health behaviors, such as alternative medical services (23). The positive association observed in this study aligns with existing findings. Additionally, existing literature indicates that the diffusion of new ideas and behaviors typically progresses from external to internal community sources, with network structural characteristics playing a significant role in this process (24, 26). This study contributes to the literature by revealing that the association between structural holes and health behavior adoption varies according to the diffusion stage. This suggests that peers with structural holes are particularly effective at promoting health behaviors in the intermediate diffusion stage.

Our findings support the structural hole theory by demonstrating associations between structural holes and health behaviors, particularly exercise and preventive dental care use. There are two possible mechanisms underlying this association. First, peers with structural holes expand their access to diverse information and perspectives, which may encourage behavioral adoption. Second, behaviors diffuse from peers with structural holes. According to the diffusion of innovation theory, early adopters are open to external information sources (24). Those with structural holes have more opportunities to access external information through connections with different networks, potentially becoming early adopters, who promote behavioral diffusion. However, this study did not measure the detailed health behaviors of peers, suggesting that further research is needed to clarify the mechanisms underlying the association between structural holes and health behaviors.

However, this study yielded unexpected results. First, the low-structural-hole category was not positively associated with exercise habits or preventive dental care use. Previous studies have shown that external sources play an important role, especially in the early stages of new behavioral diffusion (25). Because exercise habits and preventive dental care use are in the intermediate diffusion stage, we predicted that both, network members with few structural holes and those with many structural holes, would be important for adopting these behaviors. However, our findings indicate that the presence of peers with many structural holes is important for health behavior adoption not only in the early but also in the intermediate diffusion stage.

We assumed that non-smoking behavior is particularly susceptible to social influences derived from dense networks because it is in the later diffusion stage. However, peers with low levels of structural holes (i.e., many ties in the network) were not positively associated with non-smoking behavior, which is another unexpected result. There are two possible reasons for this finding. First, smoking among peers may be a confounder. Smokers are known to form clusters with each other (4). In fact, in this study, the smoking percentage among peers in the low-structural-hole group was 38%, which was higher than that of the middle- (33%) and high-structural-hole category (29%), indicating that smokers have more social ties within the network. Additionally, when one’s peers are smokers, the likelihood of the ego being a smoker increases (10). However, further adjustments for smoking among peers did not notably alter the results (data not shown). Second, dense and sparse ties may function complementarily to promote non-smoking behavior. Organizational research studies have shown that a balance between dense ties facilitating information sharing and sparse ties providing access to resources is crucial to improving organizational performance (45, 46). Similarly, in this study, peers with moderate structural holes may facilitate non-smoking behavior through a complementary effect. This suggests that a moderate level of structural holes facilitates both normative reinforcement through clustered or redundant ties and access to diverse perspectives via bridging ties. Although the results were contrary to our expectations, our findings suggest a potential role for structural holes in promoting non-smoking behavior.

Interestingly, individuals who did not report their income were more likely to engage in regular exercise and non-smoking behavior. One possible explanation is that some individuals with high income may choose not to disclose their income due to strong privacy concerns. While some individuals in low-income groups may also refrain from reporting their income due to embarrassment, their proportion within the income non-response category may be relatively small. Taken together, it is possible that individuals in the income-missing category tend to exhibit healthier behaviors due to health habits associated with higher income. However, this interpretation should be viewed with caution, as the income nonresponse group may also include individuals with other unmeasured characteristics.

The findings indicate that interventions to promote health behaviors may be effective when considering network structures according to the diffusion stage. For instance, interventions to promote behaviors at moderate levels of diffusion could foster connections between different groups, such as neighborhoods, schools, and workplaces, thereby creating new social connections. Additionally, interventions that leverage existing networks can be effective. For example, interventions encouraging individuals with structural holes to adopt the behavior could initiate further diffusion.

Our study has several limitations. First, as the study design was cross-sectional, we could not determine the causal direction of the observed associations, or assess the dynamic aspects of these constructs. For instance, because we could not clarify the stage at which behavior was adopted by egos in the diffusion process or account for health behaviors in the early diffusion stage, the association between structural holes and diffusion behavior may yet have to be fully assessed. Further, although we adjusted for various covariates, other potential confounders may have been present, such as the health behaviors of family members or relatives. Future research should use longitudinal designs to examine these mechanisms and causal directions in more detail. Second, limiting the number of peers may have resulted in insufficient representation of the ego’s intimate peer network structure. The number of peers measured varied across studies (23, 34, 47). One study recommended at least five peers to observe network effects (48). However, in egocentric network research, questions about peers can impose a cognitive load on respondents; therefore, limiting the number of peers alleviates this burden and allows more information to be obtained (36). Third, each peer’s social characteristics, health behaviors, and connections were measured based on the ego’s self-reports, making them susceptible to self-report bias. However, the ego’s perception of peer influence shapes actual behavioral choices (44). Thus, the presence of self-report bias did not negate the interpretation of our findings. Fourth, the possibility of selection bias due to missing data on smoking status cannot be ruled out. The missing rate for this variable was relatively higher than for other variables, potentially affecting the observed associations related to non-smoking behavior. Egos with missing smoking status were more likely to have peers in the low structural holes category. If smokers were more likely to withhold their smoking status, the prevalence of non-smoking behavior in this category may have been overestimated, which could have led to an underestimation of the observed negative association between non-smoking behavior and having peers with low levels of structural holes. Fifth, we assessed the structural holes of each peer but not across the ego’s entire personal network. Egos whose peers with few structural holes tended to have few structural holes in their entire personal networks. Many of these egos were in dense networks (data not shown). However, while networks with few structural holes were observed to be dense in this study, this may not necessarily apply to other networks, such as sociocentric networks. Future research should consider other structural indicators in addition to structural holes to better assess network sparsity and density. Sixth, the measurement of this study did not clearly distinguish between preventive and curative purposes of participants’ dental care use, which may lead to an overestimation of the prevalence of preventive dental care use. In Japan, it has been shown that preventive dental care is less popular than curative dental care (49). Moreover, the analytic sample may have slightly overrepresented individuals who utilize preventive dental care, as the prevalence of such use was marginally higher among egos who named four peers than those who named fewer. This may have led to a slight underestimation of the association between structural holes and preventive dental care use, as structural holes are thought to be more strongly associated with behavioral adoption in populations where the behavior is less prevalent. Seventh, our sample was collected from only four municipalities in the greater metropolitan areas of Japan, which limits the generalizability of our findings. Finally, the results of the Hosmer–Lemeshow test and pseudo R^2^ statistics suggest that the explanatory power of the models was limited, particularly for preventive dental care and exercise. This implies that other unmeasured factors may be associated with these behaviors.

Despite these limitations, this study’s novel contribution was to demonstrate that the association between structural holes and the adoption of health behaviors may differ according to the stage of diffusion of behaviors. Future studies should investigate, using longitudinal data, whether our findings apply to other health behaviors, such as alcohol use and obesity. Additionally, further investigation is needed regarding the association between health behavior adoption and other structural network characteristics, such as centrality indices, centralization, and clustering coefficient. Moreover, the heterogeneity in the associations between structural holes and health behaviors based on the nature of ties, such as relationship direction, interaction frequency, geographical proximity, relationship duration, order of nomination, and relationship type, deserves further investigation. Additionally, agent-based models could be used to simulate how variations in tie strength and network structure influence the diffusion of health behaviors, representing another promising direction for future research.

In conclusion, the present study suggests that social connections with individuals with structural holes are associated with health behavior adoption, with the association varying by the diffusion stage. These findings imply that considering social connections with different levels of structural holes according to the stage of diffusion of the target behavior may be effective for public health interventions.

## Data Availability

The data analyzed in this study is subject to the following licenses/restrictions: data are available from the Data Management Committee of the Japanese Study on Stratification, Health, Income, and Neighborhood (J-SHINE) for researchers who meet the data access criteria. Requests to access these datasets should be directed to Yasutaka Kuribayashi, bm102029@m.u-tokyo.ac.jp.

## Supplementary material

SUPPLEMENTARY FIGURE S1 Distribution of the dyad constraint index. The constraint index was calculated from dyadic constraints between the ego and each peer. Due to its moderately right-skewed distribution, the index was categorized into tertiles (low, middle, high) for analyses.
