# Supplementary figures and images for: Associations between structural holes in personal networks and health behaviors among young and middle-aged adults in Japan: a population-based cross-sectional study
Source: Front Public Health. 2025 Sep 3;13:1621420. doi: 10.3389/fpubh.2025.1621420 (PMC12440896; doi:10.3389/fpubh.2025.1621420)

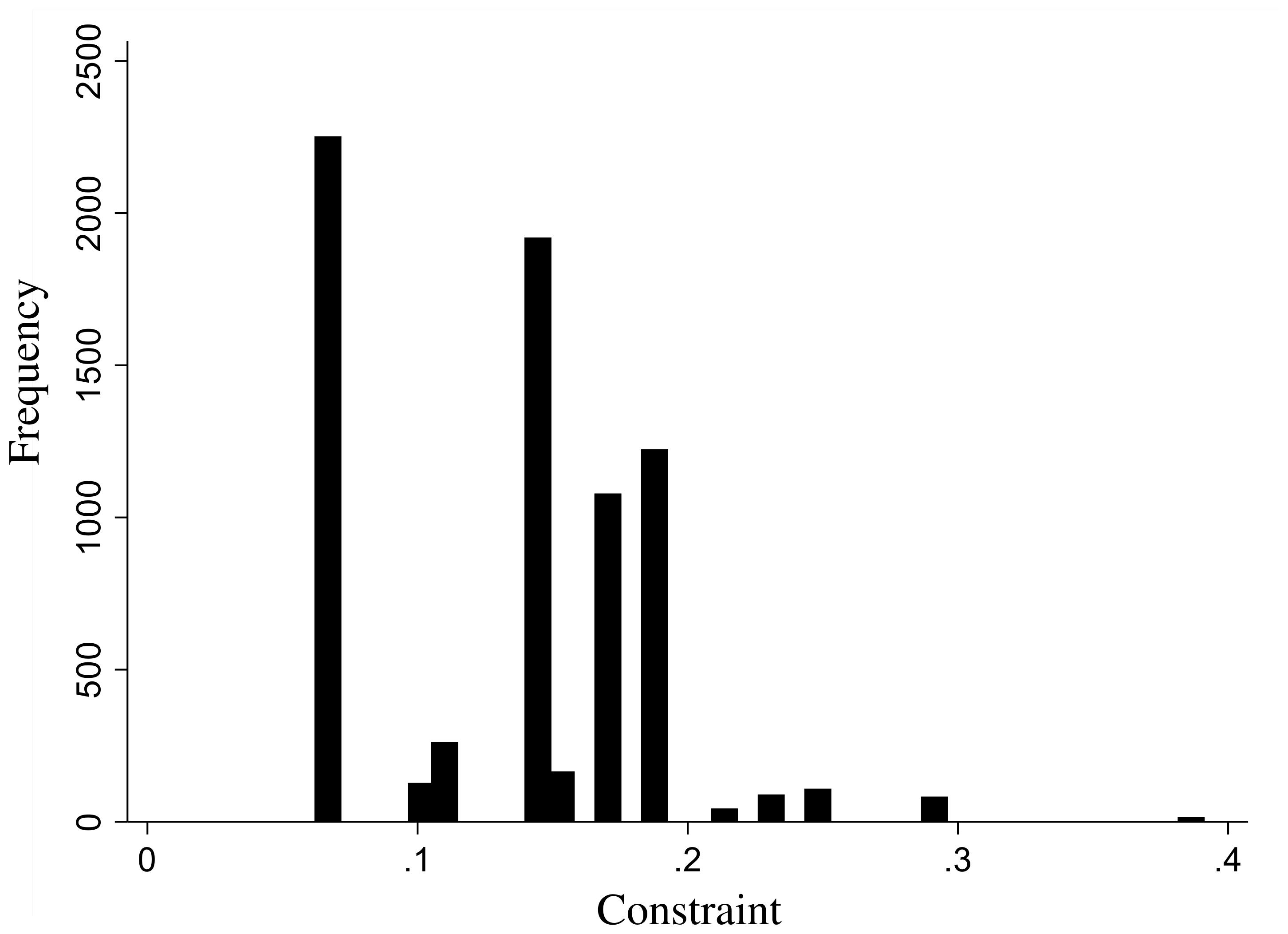

Supplement: SUPPLEMENTARY FIGURE S1 — Distribution of the dyad constraint index. The constraint index was calculated from dyadic constraints between the ego and each peer. Due to its moderately right-skewed distribution, the index was categorized into tertiles (low, middle, high) for analyses. [file Image_1.tiff]
