# Supplementary material for: Associations between structural holes in personal networks and health behaviors among young and middle-aged adults in Japan: a population-based cross-sectional study
Source: Front Public Health. 2025 Sep 3;13:1621420. doi: 10.3389/fpubh.2025.1621420 (PMC12440896; doi:10.3389/fpubh.2025.1621420)
Supplement: Supplementary file 2 [file Table_1.docx]

**Supplementary Table 1. Comparison of characteristics between egos who named four peers and those who named fewer than four.**

|  | **Egos who named**  **four peers** | | **Egos who named fewer than four peers** | |  |
| --- | --- | --- | --- | --- | --- |
|  | n = 1705 | | n = 542 | | *p*-value |
|  | n | % | n | % |  |
| Age (years), mean (SD) | 45.7 | 6.9 | 45.0 | 7.3 | 0.05 |
| Male | 716 | 42 | 228 | 42 | 1.00 |
| Educational attainment |  |  |  |  | 0.66 |
| High-school graduation or lower | 444 | 26 | 147 | 27 |  |
| College graduation or higher | 1261 | 74 | 395 | 73 |  |
| Marital status |  |  |  |  | 0.03 |
| Married | 1261 | 74 | 375 | 69 |  |
| Unmarried | 444 | 26 | 167 | 31 |  |
| Work status |  |  |  |  | < 0.01 |
| Working | 1483 | 87 | 444 | 82 |  |
| Not working | 222 | 13 | 99 | 18 |  |
| Exercise habit |  |  |  |  | 0.24 |
| At least once per week | 648 | 38 | 204 | 31 |  |
| Less than once per week | 1057 | 62 | 376 | 69 |  |
| Preventive dental care use | 870 | 51 | 249 | 46 | 0.05 |
| Smoking |  |  |  |  | 0.84 |
| Current | 245 | 13 | 76 | 14 |  |
| Former | 422 | 23 | 141 | 26 |  |
| Never | 1038 | 56 | 325 | 60 |  |
| Alcohol consumption |  |  |  |  | < 0.01 |
| 3–4 times per week or more | 631 | 37 | 115 | 27 |  |
| 1–2 times per week or less | 1074 | 63 | 309 | 73 |  |
| Equivalent income |  |  |  |  |  |
| thousand JPY (/year), | 391 | 306 | 360 | 363 | 0.03 |
| (median [IQR]) |  |  |  |  |  |
| Equivalent income categories |  |  |  |  |  |
| Low | 529 | 31 | 184 | 34 |  |
| Middle | 529 | 31 | 145 | 27 |  |
| High | 426 | 25 | 133 | 24 |  |
| DK / NA | 221 | 13 | 80 | 15 |  |
| Place of residence |  |  |  |  | 0.64 |
| Adachi | 324 | 19 | 108 | 20 |  |
| Mitaka | 357 | 21 | 119 | 22 |  |
| Kashiwa | 512 | 30 | 168 | 31 |  |
| Tokorozawa | 512 | 30 | 147 | 27 |  |

Data are presented as n (%) otherwise indicated.

Abbreviations: SD, standard deviation; IQR, interquartile range; JPY, Japanese Yen; DK/NA, Do not know/no answer.
