# Supplementary material for: Associations between structural holes in personal networks and health behaviors among young and middle-aged adults in Japan: a population-based cross-sectional study
Source: Front Public Health. 2025 Sep 3;13:1621420. doi: 10.3389/fpubh.2025.1621420 (PMC12440896; doi:10.3389/fpubh.2025.1621420)
Supplement: Supplementary file 3 [file Table_2.docx]

**Supplementary Table 2. Sensitivity analysis: logistic regression estimates using two definitions of exercise habit.**

| Exercise habit definitions: | three to four times per week or more | | a few times per month or more | |
| --- | --- | --- | --- | --- |
| Explanatory variables | Odds Ratio | 95% CI | Odds Ratio | 95% CI |
| Age | 1.04* | 1.03–1.05 | 1.02* | 1.01–1.03 |
| Male sex | 1.46* | 1.23–1.69 | 1.90* | 1.70–2.13 |
| College graduation or higher | 0.85 | 0.73–0.99 | 1.29* | 1.14–1.45 |
| Married | 1.00* | 1.00–1.00 | 1.19* | 1.06–1.33 |
| Working | 0.84 | 0.69–1.02 | 0.83* | 0.68–0.88 |
| Current smoker | 0.57* | 0.47–0.69 | 0.78* | 0.68–0.88 |
| Alcohol consumption | 1.12* | 0.98-1.29 | 1.14* | 1.02-1.27 |
| Equivalent income |  |  |  |  |
| 1st tertile | 1.00 |  | 1.00 |  |
| (Low) |  |  |  |  |
| 2nd tertile | 0.84* | 0.71–0.98 | 1.06* | 0.94–1.20 |
| (Middle) |  |  |  |  |
| 3rd tertile | 1.06 | 0.90–1.26 | 1.65* | 1.44–1.89 |
| (High) |  |  |  |  |
| Missing | 0.85 | 0.68–1.05 | 1.30* | 1.11–1.53 |
| Peer health behavior score | 1.30* | 1.08–1.55 | 1.45* | 1.27–1.65 |
| Place of residence |  |  |  |  |
| Adachi | 1.00 |  | 1.00 |  |
| Mitaka | 1.13 | 0.93–1.38 | 1.22* | 1.04–1.42 |
| Kashiwa | 1.19 | 0.99–1.42 | 1.32* | 1.14–1.51 |
| Tokorozawa | 0.92 | 0.77–1.11 | 1.16 | 1.00–1.33 |
| Structural holes |  |  |  |  |
| 1st tertile | 0.95 | 0.81–1.11 | 0.82 | 0.73–0.92 |
| (Low) |  |  |  |  |
| 2nd tertile | 1.00 |  | 1.00 |  |
| (Middle) |  |  |  |  |
| 3rd tertile | 1.40* | 1.20–1.62 | 1.21* | 1.07–1.36 |
| (High) |  |  |  |  |

95% CIs were based on robust standard errors.

Abbreviations: CI, Confidence interval.

**p* < 0.05.
